# Observations on Italian Medical Institutions and Practice; a Brief Comparative Sketch of Some Points of French and English Practice, and Remarks on Italian Climate

**Published:** 1831-12

**Authors:** Edwin Lee

**Affiliations:** Member of the Royal College of Surgeons, and Formerly House Surgeon to St. George's Hospital.


### ITALIAN MEDICAL INSTITUTIONS AND PRACTICE.

*Observations on Italian Medical Institutions and Practice; a brief Comparative Sketch of some Points of French and English Practice, and Remarks on Italian Climate.* By Edwin Lee, Esq., Member of the Royal College of Surgeons, and formerly House Surgeon to St. George's Hospital.

(Concluded from page 394.)

### ROME.

The profession is at a lower ebb in the Roman states than elsewhere in Italy: the hospitals are in a shameful state of neglect, and the mortality among poor patients is immense. Intermittent and malignant fevers from malaria are endemic in the summer. The enervating effect of the climate, the physical and moral habits of the Romans, further predispose them to attacks of fever. Dr. Clark observes, that foreigners are less liable to be affected by the malaria during the first and second years of their residence in Rome, than in subsequent years. The principal proximate cause is considered to be the impression of humidity on the skin after sunset: hence the wearing of flannel is one of the best preservatives. The insalubrity of the season is always in a direct ratio to the intensity of the heat, and the quantity of rain that has fallen. Of late years, since the improved drainage of the Pontine marshes, the frequency and severity of these fevers have diminished. There is reason to believe that the influence of malaria may remain latent for some time in the constitution, and be the occasion of various diseases at a future period. I heard of two instances, one of total, the other of partial paralysis of the limbs, occurring in young Englishmen after their return to England, which could be pretty clearly traced to this cause.

Apoplexy, or, as the Italians term it, "*accidente*," is of very frequent occurrence at Rome. Nervous affections are also very general, particularly the morbid sensibility of the olfactory nerves with respect to agreeable perfumes, which Dr. JOHNSON, in his recent work on "Change of Air," attributes to the olfactory nerves of the Romans being so habituated to the stink of their streets, as to render them unaccustomed to decent smells, and to throw them into convulsions on contact with an agreeable perfume: the dislike of perfumes is, however, by no means general among the Romans.

Acute inflammation of the lungs occasions a great mortality every winter. Rheumatism and diseases of the eyes, although prevalent, are less general than at Florence and Naples. Bronchocele is not often met with; gastric irritation, and visceral "engorgement" are of frequent occurrence. Phthisis is not frequent, except as consequent on attacks of acute inflammation.

The practice in the treatment of disease inclines to the Broussaian. The physician prescribes for any constitutional disturbance attending a surgical disease; the surgeon confining himself to operations and the application of external remedies. The abstraction of blood, in small quantities, is resorted to in the majority of cases, and as a preventive against malaria fever. Blisters are used occasionally to combat chronic inflammation. The practice of exhibiting large doses of antimony in acute inflammations is less frequently employed than formerly; the prussic acid and sedatives are frequently employed in bronchial affections. Vaccination is not encouraged; the stethoscope is not used.

The largest hospital in Rome is the *Santo Spirito,* for the reception of acute diseases: it can contain 1400 beds: at the time I was in Rome (February,) but 500 were occupied, and those chiefly on the ground floor. The building is low, but of great length; the wards are lofty, but badly ventilated and lighted, the windows being small, and placed near the ceiling. The patients are crowded together; the beds being arranged along the wards in double, and in some parts treble rows, the foot of the first bed touching the head of the second. The floors and bed furniture are dirty, and a stranger, on entering the wards, feels great inconvenience from the combination of close air and bad smells. There is a small surgical ward, containing about forty patients: the visits and dressings are made in a hurried and slovenly manner. Within the hospital are a large handsome theatre, for the delivery of anatomical and other lectures, a dissecting room, and a cabinet of pathological anatomy.

Six physicians, and I believe one surgeon, are attached to the service of this hospital. Intermitting fevers are treated by the preparations of cinchona; inflammations of the thorax, by bleeding in small quantities, the administration of antimony, and occasional blistering; in gastric or enteritic inflammation, bleeding, application of leeches, and the employment of laxatives and enematæ, are resorted to; in rheumatic affections, bleeding, warm baths, antimony, and other diaphoretics, are employed: the colchicum is not used. Separate wards are appropriated to phthisical patients; rooms are also set apart for the accommodation of patients who contribute towards their maintenance. Adjoining the hospital is an establishment for the insane.

The *Spedale St. Giacomo* is situated on the Corso, in the heart of the city, and is appropriated to chronic and surgical diseases, and to the performance of operations. The number of beds is about 350: the men are placed on the ground floor, in close and dirty wards, the beds being placed in double rows, as at Santo Spirito; the women's wards are on the first floor, and somewhat cleaner and more airy than the men's. Very little internal treatment is employed in surgical diseases beyond small bleedings, the exhibition of cooling drinks, an occasional laxative, or opiate. Almost the only dressings employed are simple charpie and emollient poultices. The mortality after accidents and operations is very great, from the frequent supervention of hospital gangrene. Ulcers and slight wounds are also very apt to run into gangrene. The lateral operation is usually performed for the stone. The cure of aneurism is attempted by compression, placing the patient on low diet, and the administration of digitalis: if these means fail, the artery is tied; in popliteal aneurism in the middle of the thigh, ligatures of reserve being used. Hydrocele is treated by injection of wine; couching is the operation usually adopted in cataract.

The *Spedale della Consolatione*, situate near the former, and in better air than the other, consists of two wards on the ground floor, on opposite sides of the street: it is exclusively appropriated to the reception of accidents. The men's ward contains about sixty beds, is very clean, and well ventilated; the patients are not crowded together, as in the other hospitals. The women's ward contains about thirty beds. Two surgeons and one physician are attached to this hospital.

Union by the first intention is attempted in most wounds. Fractures of the thigh and leg are treated as at Florence, in the extended position, and placing the limb between two ferulæ, or straight splints, connected together in the manner of a junk, by tapes passed round the limb.

Rome also contains an hospital, with 400 beds, for women labouring under acute diseases; an hospital, with 150 beds, for diseases of the skin; a lying-in and foundling hospital; and one for convalescent patients.

I shall here insert a few cases from the Clinique of Prof. Sisco, surgeon to the Spedale St. Giacomo.

*Foreign Body in the Bladder.* A man, ætat. forty-eight, had been for some time in the habit of passing a piece of straw down his urethra: on one occasion the straw broke, and a portion slipped into the bladder; this became incrusted with earthy matter, and occasioned severe pain; increased every time he passed his urine; at length he came to the hospital to have the operation of lithotomy performed, without, however, stating the original cause of his complaint. The lateral operation was performed, and the foreign body extracted with some difficulty, owing to its form. On the nature of the nucleus being ascertained, the patient confessed that the straw accidently slipped into the bladder five months previous to his admission. An opiate was given after the operation; no dressing was applied to the wound. The patient recovered, and was dismissed on the twenty-second day from the performance of the operation.

*Lithotomy in a Female.* A woman, æt. sixty, had laboured under symptoms of stone in the bladder twelve months before her admission to the hospital. The existence of a calculus having been ascertained by sounding, a grooved director was introduced into the bladder; the urethra was then divided as far as the neck of the bladder, which admited of free dilatation; forceps were introduced, but the calculus not being readily felt, these were withdrawn, and a small scoop passed; by means of this instrument the stone was brought to the neck of the bladder, and readily extracted. The calculus was of the size of a small walnut. A sedative was given after the operation. On the second day, the patient, being somewhat feverish, was bled. After a few days, an elastic gum catheter was passed into the bladder, but was removed on account of the irritation it occasioned. The patient recovered without experiencing the inconvenience of incontinence of urine.

*Compound Fracture of Cranium.* A robust young man, æt. nineteen, received two wounds on the head, in consequence of a heap of bricks falling on him: one wound was over the occiput, exposing the bone and detaching the scalp to some extent; the other was over the left parietal bone, which was fractured, and a portion depressed. The patient was stunned at the time of the accident, but, when admitted into the hospital, had no symptoms of injury of the brain. The edges of the wound of the occiput were brought in contact by adhesive plaster; the wound over the parietal bone was dressed with charpie, and a bandage applied to the head; the patient was ordered the lowest diet, and ℥xii. of blood were abstracted from the arm. No unfavorable symptoms came on.

After four days, on the dressings being removed, the wound over the occipital bone was found to be healed, and the other in a state of healthy suppuration.

Light dressings were continued; the wound healed after a small portion of bone had come away; and the patient left the hospital six weeks after admission.

*Disease of Testicle.* A man, æt. twenty-three, after the suppression of the discharge of a gonorrhœa, became affected with pain, hardness, and swelling of the right testicle. The remedies employed did not relieve him, and his surgeon intended to perform the operation of castration. This was prevented by the patient being attacked by intermitting fever, which lasted several months. On recovering from the fever, the size of the diseased testicle had increased, the patient had frequent lancinating pains in the part, and the scrotum was ulcerated. He was admitted to the hospital, and on the day following an incision was made, exposing the spermatic cord, and extending to the bottom of the scrotum. A ligature was passed under the cord, and tied tightly. The tumor sloughed in five days, and was separated by scissors. The wound was dressed throughout the cure with charpie, and the patient dismissed six weeks from his admission.

A man, æt. thirty-eight, had long been afflicted with sarcocele, which the treatment employed had not benefited; he was obliged to go into the country, and there consulted a surgeon, who, considering the disease to be hydrocele, plunged a trocar into the tumor: as no fluid escaped, he made an incision through the scrotum, and lacerated with his finger its connexions with the tumor, which occasioned an increase in its size, and considerable constitutional disturbance to the patient. The surgeon then passed a seton through its substance; the patient became worse, returned to Rome, and was admitted into the hospital. The tumor was of the size of a small melon, uneven and tuberculated on its surface, and discharging, through the apertures made by the seton, dark fetid matter. The patient was greatly reduced, with irritable pulse, and troubled with diarrhœa. After three days' rest, a bistoury was plunged into the tumor, which was laid open in its whole extent: about a pound and a half of fungous substance of a livid colour was excised, the operator fearing to remove the whole tumor, on account of the weak state of the patient. The bleeding was restrained by application of ice.

On the seventh day from the operation, a ligature was tied tightly round the base of the tumor, which separated in five days. The patient gradually recovered, and was quite well at the expiration of seven weeks.

A man, æt. twenty-two, had had disease of the testicle, which was situated in the groin, whence it had never descended to the scrotum. The tumor was of the size of a lemon, hard at its base, with a feeling of fluctuation at other parts; it was punctured with a trocar, and a quantity of fluid escaped. On the day following, the spermatic cord was exposed by an incision, and a ligature passed under it, tied tightly. Mortification of the testicle soon came on: in a few days, however, the patient experienced some stiffness in the motions of the lower jaw; tetanus supervened, and he died.

*Popliteal Aneurism.* A man, æt. thirty-six, in pursuing his adversary, exerted himself very much; a few days after, he discovered a swelling in his left ham, which quickly increased to the size of a hen's egg. On admission to the hospital, it was ascertained to be popliteal aneurism. The cure was attempted by restricting the patient to low diet, giving the digitalis, and compressing the femoral artery at its upper third by a semicircular spring, pressing on the artery and opposite side of the thigh. This treatment was continued some days, but did not affect the size of the tumor, to which ice was applied, without advantage resulting. It was then proposed to the patient to have the femoral artery tied, or the limb amputated: the patient would only submit to the amputation, which was consequently performed. It appears that he wished to die, and thought he should sink under the amputation; being disappointed in this, the same night he swallowed a quantity of opium, which he had concealed, and died the following morning.

### NAPLES.

Naples is built along part of the bay of that name, and partly on the acclivity of a hill; contains 380,000 inhabitants. The most prevalent diseases are, inflammations of the lungs, pleuritis, bronchial affections, rheumatism, gastric fevers, and diseases of the eyes.

The *Spedale degl' Incurabili* is the largest hospital: it is situated in an elevated and airy position, in the centre of the city, and can contain 1400 beds. The building is two stories high, and built round a courtyard; the wards are long and lofty, clean, and pretty well ventilated. This hospital is properly for the reception of chronic and surgical diseases; many acute diseases are, however, admitted.

The lectures of the Colleges of Medicine and Surgery on the several branches of medicine and surgery are here delivered; clinical lectures on medical and surgical cases, and on diseases of the eyes, are also given. Dr. Quadri is professor of this last branch of surgery: he employs depletion and warm applications; only at the commencement of acute ophthalmia; in the varieties of chronic inflammation, he has recourse to counter-irritation and stimulating collyria.

As at other Italian hospitals, some rooms are set apart for patients, who pay from four to six *carlini* daily. Syphilitic and consumptive patients have also wards separate from the others. There is also an incurable ward, to which moribund patients, or those considered past recovery, are transferred. Female patients are attended on by "sœurs de la charité; the men by "infirmiers," or infirmary men; the medical service is performed by twenty physicians and fourteen surgeons. Daily visits are made at an early hour.

The practice is chiefly "Hippocratique," the administration of remedies being determined by observation of the symptoms in each particular case. Bleeding is not so general as at Florence and Rome; antimony and James's powder are in very general use in acute disease: this remedy, however, is not given in the same large doses as formerly. In syphilitic cases, mercurial frictions are made in the sole of the foot, and continued for about twenty minutes each time, by an assistant, whose hand is covered by a leathern glove. Vital operations are only performed in spring and autumn, except in cases of emergency. Over the operating theatre a hand is painted, with an eye in the palm.

All the patients here, as in most continental hospitals, are confined to bed until the completion of their cure: none are seen walking about the wards.

The dressings made use of to wounds are simple, stimulating applications being rarely used; union by the first intention is attempted, where practicable.

*Case of Pneumonia and Typhoid Fever.* A man, æt. fifty-five, was received into the hospital, labouring under fever, accompanied by great prostration of strength, fixed pain in left side of thorax, great difficulty in breathing, expectoration of bloody mucus, anxious expression of countenance, and brownish dry tongue. He was ordered venesection to the amount of fourteen ounces; leeches to the side; ℥i. Ol. Ricini; and a mixture containing nitrate of potass. The thoracic symptoms were much relieved by these means: the patient was, however, greatly weakened, and had dry, arid, brown tongue.

On the third day, he was ordered a blister to the side, and a mixture containing tartarized antimony.

On the eighth day, he was more weak, and had occasional hiccup. Same medicine continued.

On the tenth day, two or three lumbrici were passed from the bowels, with several copious evacuations of fetid matter. He continued to get worse; delirium supervened, and he died on the twelfth day from his admission.

On examining the body, traces of inflammation were apparent in the stomach and ileum; the pleura was strongly adhering on left side; and small abscesses were found in left lung, which was in part hepatized.

*Dropsy, with Hypertrophy of the Heart.* A keeper of a cook-shop, æt. forty-five, was admitted, with ascites, edema of lower extremities, and great difficulty of breathing; he had laboured under dyspnœa for several months past; the pulse was, however, regular; he had no cough nor thirst, and passed his urine freely.

The means of relief employed were, the repeated application of leeches to the anus; giving the patient supertartrate of potass every morning, two grains of Pulv. Scillæ every night, and putting him on milk diet: the disease, however, made progress. The diuretics were varied, and the digitalis given, but without benefit; and the patient died fifteen days after his admission. The diagnosis on his admission was hydrothorax, followed by abdominal and serous effusion.

On examination post mortem, the peritoneum and intestines were healthy; several quarts of fluid in the abdomen; liver enlarged and tuberculated; extensive adhesions of the pleuræ; lungs slightly inflamed; the heart much increased in size, and softened in its substance; ascending aorta greatly dilated. The quantity of serum found in the thorax was less than had been anticipated.

*Acute Ascites.* A porter, æt. thirty-two, who had enjoyed good general health, was admitted into the hospital, with ascites and anasarcous lower extremities. The abdomen was not much distended, but fluid was readily felt on percussion. He first felt himself indisposed after exposure to wet and cold, a fortnight before his admission.

On admission, he had thirst and dry skin; the tongue was redder than natural; pulse regular, but hard; bowels constipated; urine scanty. He was ordered milk diet, a bleeding from the arm, and saline purgatives. In a few days, a sensible amelioration had taken place; the abdomen was less tumid, and the anasarca of the lower extremities had diminished. A continuance in the same plan completed the cure, and he left the hospital three weeks after admission.

*Chronic Ascites.* The patient, a man æt. forty-five, was of an emaciated and cachectic appearance; he resided in a marshy district, and had felt, for some weeks previous to application for relief, obscure wandering pains in the abdomen; great weakness; he experienced constant thirst, and his urine was scanty. When admitted into the hospital, the existence of fluid in the abdomen was apparent on percussion; the tongue was clean, and no appearance of disease of the liver existed. The ascites was considered to be the result of chronic peritonitis, the predisposing cause of which was the malarious influence to which he had been constantly subjected. He was sent to an establishment for convalescents in the country: was allowed to walk in the open air; prescribed milk diet; ℥ss. Oxymel Scillæ night and morning. A few weeks passed over without any perceptible change for the better or worse. The quantity of Oxymel Scillæ was increased to ℥ii. per diem, and blisters were applied to different parts of the abdomen. By perseverance in this plan, he recovered firm health, and was discharged three months after application at the hospital.

*Popliteal Aneurism.* A man, æt. sixty, of good constitution, was received with an aneurism of the size of a small orange in the right ham: he had no appearance of any other disease of the vascular system. Low diet, bleedings, digitalis, and the application of cold to the tumor, were the means prescribed. This treatment was continued for some weeks, without effect. The femoral artery was then tied in the upper third of the thigh; a ligature of reserve was employed. The pulsation in the tumor ceased, and all went on well for some days, when the pulse became more frequent, and occasionally intermitted. A bleeding from the arm was practised, and cooling drinks ordered. In a few days the pulse became more weak, and cough, dyspnœa, and spitting of blood supervened; the bleeding was repeated, and hyoscyamus given: the pulmonic symptoms yielded; the patient recovered his strength, and, being cured of the aneurism, was dismissed in three months.

In the composition of the notes on Bologna, Parma, and Padua, I have extracted largely from the work of Dr. Valentin;* as the time I remained in those places was too short to allow me to visit the medical institutions, except in a very cursory manner.

### BOLOGNA,

Situate in an extensive plain at the base of the Appenines, was the headquarters of the theory of contra-stimulus. Dr. Tommasini was until lately professor of clinical medicine to the university. The number of students is between five and six hundred annually. The university contains theatres for different lectures, dissecting rooms, chemical laboratory, and anatomical wax models. Galvani, Malpighi, and Valsalva, were educated at this university.

Dr. Clark, in his "Notes," gives the plan of study adopted in the following order: Medical students are obliged to attend

* Voyage Medical en Italie.

the classes during four years: in the first year, lectures on natural history, botany, chemistry, anatomy; second year, anatomy, physiology, comparative anatomy, institutes of surgery; third year, pathology, clinical medicine, materia medica, chemistry; fourth year, pathology, clinical medicine, medical jurisprudence, and midwifery. During the last year of study, a certain number of patients are placed under the care of each pupil, who, previous to his examination, has to give an account of the cases, and of the treatment he has adopted. Surgical students, during the first and second years, attend the same courses of lectures as the medical pupils; third year, institutes of surgery, clinical surgery, anatomy, and dissections; fourth year, medical jurisprudence, midwifery, dissections, clinical surgery, and the performance of operations on patients, under the guidance of the Professor. At the termination of the first year, students take their degree of bachelor; at the end of the second year, of licentiate; and at the end of the fourth year, of doctor of medicine or surgery.

The mode of examination of candidates is as follows: five professors of the different branches of education submit each to the candidate twenty different subjects, taken from his own course of instruction; the pupil draws one of these by lot, and is examined on that subject. Thus the candidate is examined on five subjects connected with medicine or surgery. When the examination is terminated, each of the professors gives his vote separately as to the fitness of the candidates: those who are not considered sufficiently competent have to study during another year.

The prevalent diseases of Bologna are thoracic and gastroenteritic inflammations, phthisis, rheumatism, and intermittents. In the treatment of disease, the theory of contrastimulus is more followed at Bologna than elsewhere: the followers of this doctrine consider that the regular exercise of the functions of the body is maintained by a due equilibrium being kept between the states of stimulus, or exalted excitability, and contra-stimulus, or diminished excitability; and when this equilibrium is destroyed by any cause, disease exists, requiring for its removal, in the first case, contrastimulant remedies, as bloodletting, general or local; medicines tending to diminish vascular action, as sedatives, digitalis, antimony, blistering the surface of the body, and cooling beverages. In the opposite state of diminished excitability, or contra-stimulus, remedies are prescribed, which tend to increase vascular action, as stimulants, tonics, &c.

The mortality of those attacked by inflammation of the lungs is about one in four.

The *Spedale della Vita* contains about 200 beds: the wards are airy and clean, and the service performed with care and regularity. Two physicians and two surgeons perform the professional duties of this hospital, and deliver clinical lectures to the students of the university. The hospital is appropriated to the reception of acute and surgical diseases and accidents. Intermitting fevers are treated with the sulphate of quinine, preceded by an emetic or purgative.

The *Spedale St. Orsola* is for chronic and syphilitic complaints, and also for insane persons. The nux vomica has been given at this hospital, with great success, in cases of paralysis. Poor patients are also visited at their own habitations: each parish provides a physician and a surgeon for this purpose.

PARMA,

With a population of 35,000 inhabitants, contains an hospital for medical and surgical diseases, a lying-in and foundling hospital, an establishment for the reception of the insane, and an university.

The *Spedale della Misericordia,* or general hospital, has about 350 beds: the wards are clean and airy, and great attention is paid to the comforts of the patients. There are two clinical wards, one medical and one surgical; each contains twelve patients of both sexes, selected from the most instructive cases in the hospital. Clinical discourses are held at the bedside of the patients.

The university is but thinly attended by students. Lectures on the various sciences are delivered during eight months in the year. The doctrine of contra-stimulus is followed in the treatment of disease, although less than formerly.

There is also at Parma a society for the relief of poor persons, and affording them medical assistance at their own habitations: this is called the *Congregazione Pietosa della Caritá,* and was established in the fifteenth century; one half of the members are ecclesiastics, the other half is composed of nobles and citizens. Two members of the society are attached, in rotation, to each district of the city and adjoining country, whose duty it is to seek out and relieve those persons who may need assistance. Several physicians and surgeons, who are elected every three years, perform the medical duties, and receive a salary from the society; the physicians about eighteen pounds, the surgeons eleven pounds annually. The affairs of the society are managed by twelve members, six secular, six ecclesiastic, who are divided into pairs; each pair have a particular department to superintend. Medicines, and all other articles of which the poor may stand in need, are gratuitously furnished them by the society.

### PADUA.

The population of Padua is about 25,000 souls; it contains the largest university in Italy: the number of students is estimated at about 1500 annually; many are from foreign countries; the medical classes are attended by about one third of the above number. On the wall around the court-yard are the heads in relievo, with inscriptions of the names, &c., of those celebrated men who have done honour to the university; among these is the head of Harvey. Lecture rooms, an anatomical museum, one of natural history, a library and chemical laboratory, are contained in the university. The faculty of medicine is composed of a director, a dean, and thirteen professors of the different branches of medical and surgical education. Caldani is professor of anatomy; Brera professor of clinical medicine; Ruggieri of surgery and pathology.

Padua contains two hospitals; the largest has 300 beds, and is appropriated to the reception of acute diseases. Clinical lectures are delivered by the professors of clinical medicine and surgery of the university. The other hospital is for chronic complaints. There is also at Padua a society for the relief of indigent patients.

### MILAN

Is situated on an extensive plain, bounded on the north by the Alps, and contains 120,000 inhabitants. There are three principal hospitals: the *Spedale Grande* is perhaps the largest building of the kind in Europe; its façade measures 900 feet in length; it is composed of a ground floor and first floor, built around a spacious courtyard, and contains 2000 beds. The wards are long, lofty, and well aired. Ten physicians, four surgeons, five assistant surgeons, and eight "internes," or house surgeons and physicians, perform the medical duties. The medical visit takes place daily at six o'clock; the surgical visit as soon as the physicians have completed their rounds. The average number of patients in the hospital is 1000; there are also many out-patients: all diseases are admitted. Separate wards are appropriated to those patients who pay for the extra accommodation.

The treatment of disease does not differ materially from the practice followed in the French hospitals. Fractures of the thigh are treated by the long splint; fractured legs are placed in junks; in operations for aneurism, ligatures are used composed of several threads; "*ligatures d'attente*" are employed. Union by the first intention is attempted in all incised wounds; dressings are of the simplest kind. Purgatives are more freely used at Milan than elsewhere in Italy. Vaccination is encouraged by the government, and smallpox is rarely seen.

The *Santa Catarina*, or foundling hospital, receives nearly 3000 infants annually; many of these are sent to breathe a purer air in the country.

The irrigation of the rice fields, with which the Milanese abounds, is a fertile source of fevers of all types; which, with thoracic inflammations, chronic bronchial affections, phthisis, rheumatism, and gastric irritation, form the prevalent diseases. The pellagra is endemic throughout the Milanese: it is chiefly benefited by change of air, warm baths, and generous living.

### TURIN

Contains 80,000 inhabitants. The principal hospital, *San Giovanni Battista*, for the reception of acute and chronic diseases, has about 400 beds. The physicians are elected every ten years, assistant physicians every four years: these last reside in the hospital, and receive a yearly salary of about thirty pounds. Clinical lectures are delivered.

The *Maniconico*, or establishment for the insane, is much better managed at present than when Dr. Clark published his account of the institution. Chains are never used as a means of restraint, but recourse is had to the strait-waistcoat. The indiscriminate bleeding and purging the patients every spring, formerly practised, is now discontinued. Among the remedial means now in use, where great cerebral excitement exists, are general and local bleeding, purgatives, tepid and shower baths, the application of iced cloths to the head. In many cases where these means have failed to afford relief, opium has been used with great advantage. Noisy and intractable patients are placed for a short time in a dark room, well padded, without confinement of the hands or legs: this means usually renders them quiet. In some cases of furious mania and deep melancholia, the application of the actual cautery to the nape of the neck, so as to form a deep burn, has been productive of very beneficial effect, after the failure of other means. The remedy was introduced by the recommendation of the French physician, Dr. VALENTIN.* Among the more tractable patients, various moral means are em-

* Mémoire sur l'Emploi du Cautere Actuel; 8vo. Nancy, 1815.

ployed, such as gymnastic exercises, gardening, swinging, and mechanical labour, the training of birds, knitting, spinning, &c.; the introduction of reading and music is in contemplation. The number of patients in the institution last year was about 320; the great majority were unmarried persons, and the men in greater proportion.

### *Remarks on the Italian Climate.*

It may not be out of place to offer here a few remarks on the climate of particular parts of Italy, considered in a remedial point of view. The vague notions which generally prevailed until lately in this country, respecting the benefit to be derived from a southern climate in consumptive diseases, induced many invalids to repair to the south of France and Italy, in the hope that, after the failure of other means, they had still a resource in the balmy air of the south. From want of discrimination as to the diseases likely to be benefited by climate, and in the choice of a residence, the expectations of patients and their friends were doomed, in the majority of instances, to end in disappointment, and the error was only discovered when too late to be retrieved.

Owing to the increased intercourse between England and the continent of late years, and to the able treatise of Dr. Clark on Climate, the subject is now much better understood, and more discrimination used in the selection of cases likely to derive benefit from climate. Many patients, however, unwilling to forego this last hope, annually leave England, when in a state of health little likely to be benefited by any change, who might be deterred from leaving home, and needlessly subjecting themselves to the many privations attendant on travelling and living abroad, were the subject of climate more generally understood. I have been induced to make the following remarks, thinking that any information on this important subject cannot be too universally diffused.

It is now generally admitted that the south of France, formerly resorted to by so many invalids labouring under consumptive and bronchial complaints, is one of the worst climates that could be selected for the winter residence of such individuals: in fact, the frequent prevalence of the "*vent de bise*" renders great care necessary on the part of persons in health; and inflammations of the lungs, bronchial affections, and consumption are almost as frequently met with as in England.

Many parts of Italy are liable to the same objection: the piercing *tramontana*, or north wind, is felt as severely as the "*vent de bise*" in the south of France, while at the same time, in some situations, the sun has great power, and renders the inhabitants more susceptible to inflammatory attacks of the chest and air-passages, on exposure to cold winds, than persons living in a climate uniformly cold. The greater number of Italians guard against these variations of temperature by the constant practice of wearing large cloaks, without which they seldom stir out during five months of the year: foreigners, however, who are less cautious in this respect, frequently experience the bad effects of their negligence.

The cities mostly frequented by the English during winter are Nice, Pisa, Florence, Rome, and Naples.

NICE. The sky of Nice is generally clear, and the air light, dry, and bracing; hence it is well adapted to individuals of a relaxed habit. The soil is rich in vegetable productions; the olive, pomegranate, lemon, orange, almond, fig, and vine grow luxuriantly. The climate is more settled, and the transitions of temperature less frequent, than at Pisa, Florence, or Naples. Nice has the maritime Alps behind it, by which it is sheltered from the "*vent de bise*," or north winds: easterly and north-westerly winds are, however, severely felt, particularly towards the spring, at which time pulmonary complaints are very prevalent among the inhabitants: on this account it is not calculated for the winter residence of consumptive patients. According to Dr. Clark, the mean temperature of Nice in winter is nine degrees warmer than London; in spring, seven degrees warmer.

The complaints which the climate of Nice is likely to benefit are, phthisis in the earliest stage, or where the predisposition to this disease is not fully developed, and the patient not subject to attacks of pulmonary or bronchial inflammation: such invalids should, however, not remain at Nice later than the beginning of February, when their residence may be advantageously transferred to Pisa or Rome for the remainder of the winter: the constant communication by steam between the ports of the Mediterranean will obviate the fatigue and inconvenience of travelling at this period of the year. Patients with chronic bronchial or asthmatic diseases, in whom little tendency to inflammatory action exists; nervous and hypochondriacal patients of relaxed habits, and those labouring under the chronic forms of dyspepsia, will derive great advantage from passing the winter at Nice.

PISA is built on the Arno, distant six miles from the sea, and is much frequented in winter by patients suffering under pulmonary or bronchial disease. The air is less dry and sharp than that of Nice; less soft than that of Rome; it is sheltered by hills from the full influence of the north and east winds, and is less liable to great and sudden variations of temperature than Florence and Naples: cold winds are, however, occasionally severely felt at Pisa. The north bank of the river, called Lung' Arno, where the houses have a southern aspect, is the most eligible situation for invalids. The heat of the sun is at times very great, and there exists a difference of several degrees of temperature between this situation and other parts of the city less exposed to its influence, and less sheltered from cold winds. Many patients with laryngeal and bronchial affections, particularly young persons, are permanently benefited by passing a winter at Pisa: the climate is also well suited to the majority of consumptive patients. Persons predisposed to phthisis, and in the earliest stage of this disease, will in general derive great advantage from passing two or three successive winters at Pisa: it is, however, a dull winter residence, both on account of its offering none of the resources of a capital city, and from the number of invalids there congregated. For those patients who require more amusement, Rome is preferable in many respects: many patients, however, find Pisa agree better with them than the more relaxing climate of Rome.

Florence is, perhaps, the residence least adapted to persons suffering from disease of the lungs, trachea, or bronchi, or to those liable to rheumatic complaints: the winter is generally very severe; the transitions of temperature great, frequent, and sudden. The *tramontana*, sweeping over the Apennines, is piercing and sharp; while at the same time, in some parts of the city, the heat of the sun is inconveniently felt: thus in one minute the change from the heat of summer to the cold of winter may be experienced. With some patients labouring under nervous asthma, Florence has agreed better than any other city in Italy: such persons should reside in that part of the city north of the Arno. With dyspeptic, hypochondriacal, and nervous patients, who seek mental recreation in travelling, the climate of Florence will, in general, be found not to disagree; while the city has more resources for amusement than any other in Italy.

Rome. The climate of Rome is milder, more equable, and the winter shorter and less severe, than at Pisa: the *tramontana* is, however, sometimes prevalent for several days together; during which period an invalid cannot leave the house without danger; yet, in general, the air is light, soft, and balmy. After much rain, and during the prevalence of the sirocco wind, the air is humid; at other times, less so than that of Pisa. Rome has the advantage over Pisa in possessing more resources for mental and bodily recreation, and is perhaps the best winter residence for consumptive patients in general. For those persons with bronchial affections, in whom a tendency to inflammation or irritation of the air-passages exists, Rome is the most advantageous winter residence: with some patients, however, of this class Pisa agrees better.

In the more chronic form of bronchial affection, and that called winter cough of elderly persons, the climate of Nice is preferable to either Rome or Pisa. Rheumatic and dyspeptic patients are generally benefited by wintering at Rome.

Naples. The climate of Naples is one of the most variable in Italy: cold winds are severely felt in that part of the city inhabited by foreigners, viz. the Sta. Lucia Chiaja and Strada Vittoria, and the houses are less calculated to exclude cold air than those in other Italian cities. The heat of the sun, and the relaxing effects of the sirocco wind, render invalids extremely susceptible of the great and frequent changes of temperature. All patients with thoracic, tracheal, or rheumatic complaints, should avoid Naples during the winter and early part of the spring. For nervous and hypochondriacal patients, who require mental relaxation, Naples joins to the bustle and gaiety of a large capital the advantages of clear sky, beautiful scenery, and a residence on the sea shore.

Baths of Lucca. For those who are unable or unwilling to leave Italy during the summer, the Baths of Lucca offer the most eligible and agreeable residence: the season is from the middle of May to the end of August, during which time the weather is fine and settled. It is the coolest summer residence in Italy, and possesses sheltered walks, where exercise may be taken at any hour of the day. The part called the Ponte Seraglio, and the Bagni alla Villa, are pleasantly situate in a valley among the Apennines, on a branch of the river Serchio, and distant about three quarters of a mile from each other. The most eligible situation, however, for pulmonary or rheumatic invalids is the part called the Bagni Caldi, situate on a hill overlooking the Ponte Seraglio, at which last-named place, and at the Bagni alla Villa, considerable humidity prevails towards evening. The water which supplies the baths is derived from hot springs; it has not any active medicinal properties, and is beneficial in rheumatic and other complaints.

The months of September, October, and part of November, may be passed at Florence, where, during this period, the weather is generally fine and mild. Leghorn may also be advantageously resorted to during the month of September.

On the whole, therefore, I should say that, in consumption, Pisa and Rome are the only two places in Italy likely to be beneficial in retarding the melancholy termination of the disease: in the earliest stage of phthisis, and in many bronchial complaints, permanent benefit may be derived from wintering in either of these cities; but in those cases where the existence of phthisis is manifest previous to leaving England, the friends of patients will act more considerately in counselling them to seek such alleviation as the climate of the south and south-west of England will afford, among the comforts of home, sooner than to undergo, to so little purpose, the fatigues of travelling, and the inconveniences and discomforts of a residence abroad.

---

